# Phosphorylation of Ubc9 by Cdk1 Enhances SUMOylation Activity

**DOI:** 10.1371/journal.pone.0034250

**Published:** 2012-04-03

**Authors:** Yee-Fun Su, Tsunghan Yang, Hoting Huang, Leroy F. Liu, Jaulang Hwang

**Affiliations:** 1 Molecular and Cell Biology Program, Taiwan International Graduate Program, Graduate Institute of Life Sciences, National Defense Medical Center and Institute of Molecular Biology, Academia Sinica, Taipei, Taiwan; 2 Institute of Molecular Biology, Academia Sinica, Taipei, Taiwan; 3 Department of Pharmacology, Robert Wood Johnson Medical School, University of Medicine and Dentistry of New Jersey, Piscataway, New Jersey, United States of America; University of Washington, United States of America

## Abstract

Increasing evidence has pointed to an important role of SUMOylation in cell cycle regulation, especially for M phase. In the current studies, we have obtained evidence through *in vitro* studies that the master M phase regulator CDK1/cyclin B kinase phosphorylates the SUMOylation machinery component Ubc9, leading to its enhanced SUMOylation activity. First, we show that CDK1/cyclin B, but not many other cell cycle kinases such as CDK2/cyclin E, ERK1, ERK2, PKA and JNK2/SAPK1, specifically enhances SUMOylation activity. Second, CDK1/cyclin B phosphorylates the SUMOylation machinery component Ubc9, but not SAE1/SAE2 or SUMO1. Third, CDK1/cyclin B-phosphorylated Ubc9 exhibits increased SUMOylation activity and elevated accumulation of the Ubc9-SUMO1 thioester conjugate. Fourth, CDK1/cyclin B enhances SUMOylation activity through phosphorylation of Ubc9 at serine 71. These studies demonstrate for the first time that the cell cycle-specific kinase CDK1/cyclin B phosphorylates a SUMOylation machinery component to increase its overall SUMOylation activity, suggesting that SUMOylation is part of the cell cycle program orchestrated by CDK1 through Ubc9.

## Introduction

SUMOylation, a dynamic post-translational modification process, requires E1 (SAE1/SAE2), E2 (Ubc9) and multiple E3s (e.g. Siz and PIAS in vertebrates) to carry out covalent conjugation of SUMO (e.g. SUMO1, SUMO2 and SUMO3 in mammalian cells) to target proteins, and a number of de-SUMOylation enzymes (i.e. Ulp/SENPs) for rapid deconjugation [1]. Like other post-translational modifications, SUMOylation has been shown to be involved in many cellular processes [1]. Particularly, there is increasing evidence supporting a major role of SUMOylation in mitosis [2], [3].

Studies of a temperature-sensitive mutant in budding yeast have demonstrated that Ubc9 is required for progression through mitosis [4]. Ubc9 (Hus5) mutants in fission yeast also display defects during chromosome segregation and reduced cellular growth [5], [6]. Loss of Ubc9 in mouse embryos causes chromosome mis-segregation and loss of nuclear integrity [6]. Studies in Zebra fish have also suggested an *in vivo* requirement of Ubc9 for G2/M transition and/or progression through mitosis during vertebrate organogenesis [7].

Studies of other SUMOylation machinery components have suggested a similar role of SUMOylation in cell cycle progression through mitosis. In budding yeast, temperature sensitive Smt3 (budding yeast SUMO) mutants show defects in chromosome segregation [8]. In fission yeast, pmt3Δ cells also show defects in mitotic chromosome structure or segregation errors [9]. These defects include high frequency loss of mini-chromosomes and a cut (cell untimely torn) phenotype. It has also been demonstrated that SUMO-1 targets RanGAP1 to kinetochores and mitotic spindles [10]. In addition to Ubc9 and SUMO, SUMO proteases have also been shown to play an important role in mitosis as budding yeast *ulp1* mutants show cell cycle delays at the G_2_/M boundary and elevated chromosome mis-segregation [11]. In addition to the genetic analysis, biochemical studies have also demonstrated that key chromosome structural components (i.e. condensin and cohesin complexes, and DNA topoisomerase II) are mitotic SUMOylation targets [2], [12]–[15]. Condensin and cohesin contain Structural Maintenance of Chromosomes (SMC) proteins [16]. SMC proteins such as Smc1p, Smc3p, Smc5p have been identified as SUMOylation targets in budding yeast via proteomic screens [12], [14]. topoisomerase II has been shown to be a major target of SUMOylation in both budding yeast and vertebrates [2], [13], [15]. SUMOylation of topoisomerase II has been shown to play a key role in centrosome cohesion and chromosome segregation [2], [13]. Expression of the *SUMO*-null form of Aurora B also results in abnormal chromosome segregation and cytokinesis failure [3].

Cyclin-dependent kinases (Cdks) are master regulators for cell cycle progression [17]. Cdks associate with distinct cyclins to activate its phosphorylation activity. At least 4 interphase Cdks (Cdk2, Cdk3, Cdk4 and Cdk6) are required to drive cells through interphase, while CDK1 functions primarily to drive mitosis in mammalian cells. Cyclin B is known to associate with CDK1 to drive G2/M transition and progression through mitosis, as evidenced by its involvement in chromosomal condensation, nuclear envelope breakdown, centrosome separation and mitotic exit [18]–[21]. The central role of CDK1/cyclin B in regulating mitosis has prompted us to investigate a possible link between CDK1/cyclin B and SUMOylation.

In the current studies, we have studied the possible interplay between the master mitotic kinase CDK1/cyclin B and SUMOylation. We show that CDK1/cyclin B, but not many other cell cycle kinases, enhances sumoylation activity. In addition, CDK1/cyclin B specifically phosphorylates Ubc9 at serine 71, but not other SUMO machinery components such as SAE1/SAE2 and SUMO1. Most importantly, CDK1/cyclin B-mediated phosphorylation of Ubc9 leads to elevated SUMOylation activity and stimulates the formation of the Ubc9-SUMO1 thioester conjugate. In the aggregate, these results suggest that Ubc9 phosphorylation and hence elevated SUMOylation may be part of the cell cycle regulatory program orchestrated by CDK1/cyclin B.

## Results

### CDK1/cyclin B Stimulates SUMOylation *In vitro*

**Figure 1:**
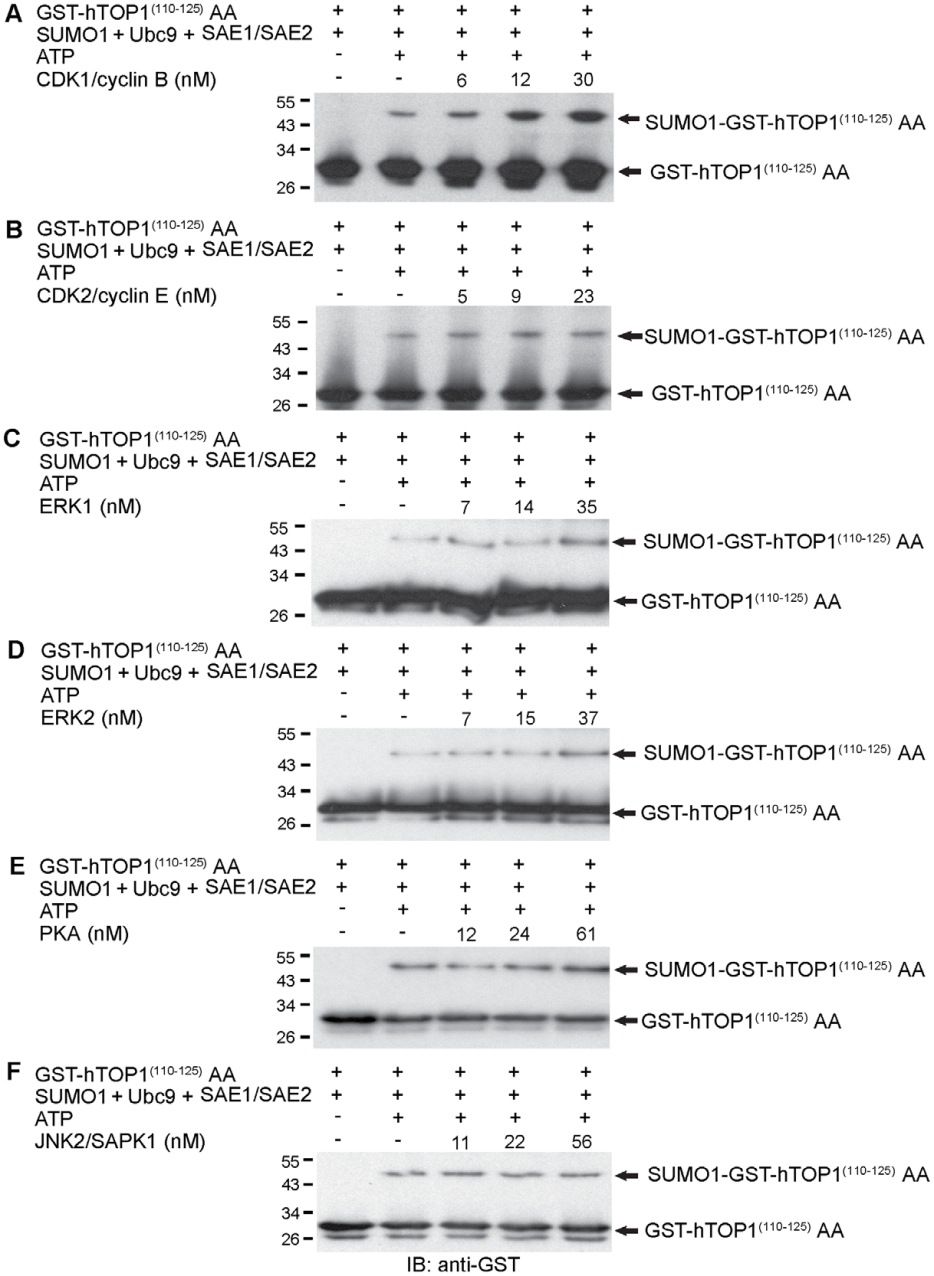
CDK1/cyclin B stimulates SUMOylation *in vitro*. (A-F) Various concentrations of different recombinant kinases, CDK1/cyclin B (6 nM, 12 nM and 30 nM), CDK2/cyclin E (5 nM, 9 nM and 23 nM), ERK1 (7 nM, 14 nM and 35 nM), ERK2 (7 nM, 15 nM and 37 nM), PKA (12 nM, 24 nM and 61 nM) and JNK2/SAPK1 (11 nM, 22 nM and 56 nM), were added to *in vitro* SUMOylation reaction mixtures containing His_6_-SUMO1, His_6_-Ubc9, SAE1/His_6_-SAE2, GST-hTOP1 ^(110–125)^ AA and ATP at 37°C for 60 min. The reactions were then boiled in SDS sample buffer and analyzed by 15% SDS-PAGE, followed by immunoblotting with anti-GST antibody.

In order to gain a better insight into the link between cell cycle progression and SUMOylation, we have tested the effect of various cell cycle kinases (i.e. CDK1/cyclin B, CDK2/cyclin E, PKA, ERK1, ERK2 and JNK2/SAPK1) on SUMOylation in an *in vitro* system (see Materials and Methods). In addition to His_6_-SUMO1, His_6_-Ubc9, SAE1/His_6_-SAE2, the *in vitro* SUMOylation system also included an artificial substrate, GST-hTOP1^(110–125)^ AA which contained a single SUMOylation motif of human topoisomerase I (hTOP1) fused with GST [22]. Serine 112 (a reported target of CDK1/cyclin B) and serine 111 of GST-hTOP1^(110–125)^ were mutated to alanine [23]. Addition of CDK1/cyclin B was shown to enhance SUMOylation in a concentration-dependent manner (**Figure 1A**). The effect of CDK1/cyclin B on SUMOylation appeared specific since many other cell cycle kinases, CDK2/cyclin E, ERK1, ERK2, PKA and JNK2/SAPK1, failed to significantly stimulate SUMOylation in the same concentration range (**Figure 1B-F**). It is noted, however, that ERK1 and ERK2 exhibited an observable but weak stimulatory activity (**Figure 1C-D**). Under our *in vitro* SUMOylation assay conditions, all kinases were able to phosphorylate their specific target proteins, indicating their normal phosphorylation activity (**Figure S1; Text S1**).

### CDK1/cyclin B Specifically Phosphorylates the SUMOylation Machinery Component Ubc9

**Figure 2:**
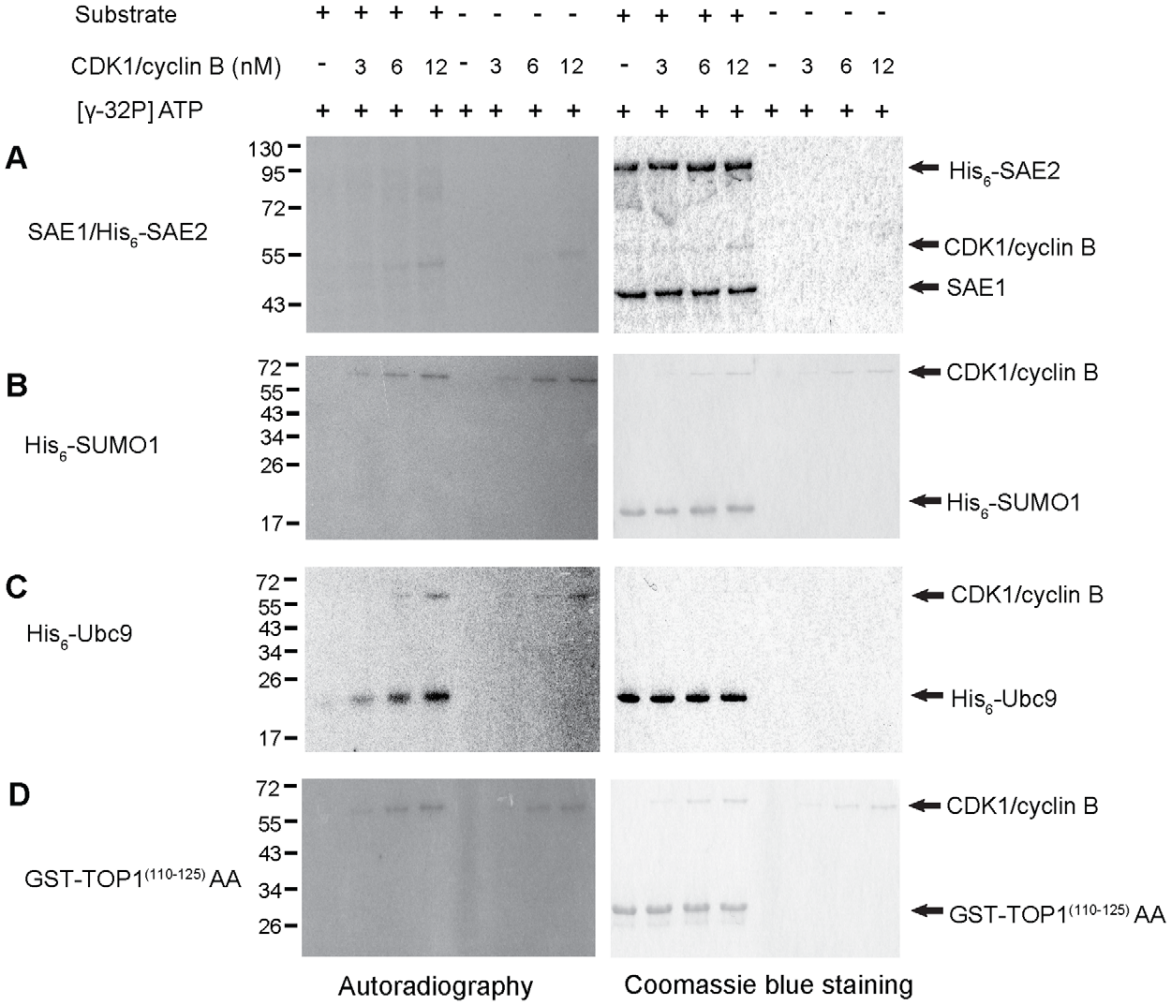
CDK1/cyclin B phosphorylates Ubc9 *in vitro*. (A) *In vitro* phosphorylation of SAE1/SAE2 by CDK1/cyclin B. CDK1/cyclin B (3 nM, 6 nM and 12 nM) was incubated with (left) or without (right) SAE1/His_6_-SAE2 for 30 min at 30°C in the presence of [γ-^32^P] ATP. The reactions were analyzed by 10% SDS-PAGE followed by Coomassie blue staining and autoradiography. (B) *In vitro* phosphorylation of His_6_-SUMO1 by CDK1/cyclin B. Various concentrations of CDK1/cyclin B (as mentioned above) were incubated with (left) or without His_6_-SUMO1 (right) in the presence of [γ-^32^P] ATP for 30 min at 30°C. (C) *In vitro* phosphorylation of Ubc9 by CDK1/cyclin B. Various concentrations of CDK1/cyclin B (as mentioned above) were incubated with (left panel) or without His_6_-Ubc9 (right panel) in the presence of [γ-^32^P] ATP for 30 min at 30°C. (D) *In vitro* phosphorylation of GST-hTOP1 ^(110–125)^AA. Various concentrations of CDK1/cyclin B (as mentioned above) were incubated with (left) or without GST-hTOP1^(110–125)^AA (right) in the presence of [γ-^32^P] ATP. Reaction mixtures of B, C and D were analyzed by 15% SDS-PAGE followed by Coomassie blue staining and autoradiography.

The stimulatory activity of CDK1/cyclin B on *in vitro* SUMOylation could possibly be related to its kinase activity. To test this possibility, the CDK1/cyclin B-mediated phosphorylation of the SUMO machinery components (SAE1/SAE2, SUMO1 and/or Ubc9) was investigated *in vitro* in the presence of [γ-^32^P] ATP (see Methods and Materials). As shown in **Figure 2A**, CDK1/cyclin B was shown to cause an observable, but relatively weak, increase in the radio-labeling of His_6_-SAE1 and His_6_-SAE2 (**Figure 2A**). However, the radio-labeling exhibited a low background in the absence of CDK1/cyclin B. CDK1/cyclin B did not cause any detectable phosphorylation of His_6_-SUMO1 in the same CDK1/cyclin B concentration range (**Figure 2B**). By contrast, CDK1/cyclin B caused a concentration-dependent increase of phosphorylated His_6_-Ubc9 as evidenced by the appearance of a radio-labeled band (revealed by autoradiography) with the same mobility as His_6_-Ubc9 band (as revealed by the corresponding coomassie-stained band) (**Figure 2C**). On the other hand, SUMOylatable substrate GST-hTOP1^(110–125)^AA was unaffected by the stimulatory effect of CDK1/cyclin B (**Figure 2D**). Histone H1, a known CDKs target protein, was strongly phosphorylated by CDK1/cyclin B, illustrating the normal activity of CDK1/cyclin B (**Figure S2; Text S1**). Collectively, these observations suggest that Ubc9 is the only SUMO machinery component to be significantly phosphorylated by CDK1/cyclin B.

### CDK1/cyclin B-phosphorylated Ubc9 Exhibits Elevated SUMOylation Activity

**Figure 3:**
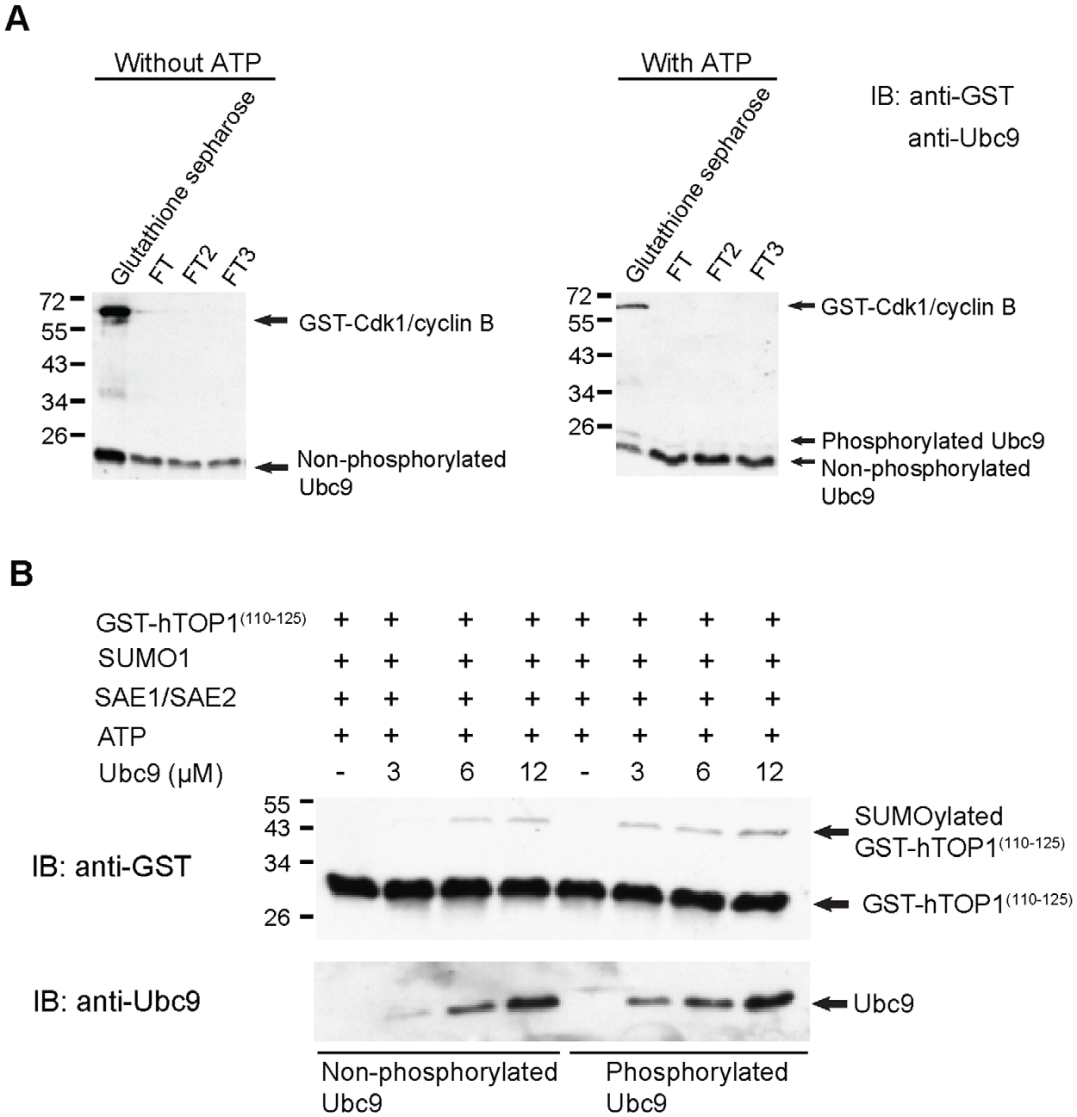
CDK1/cyclin B-phosphorylated Ubc9 exhibits elevated SUMOylation activity. (A) Purified His_6_-Ubc9 was incubated with GST-CDK1/cyclin B in the presence or absence of ATP for 30 min at 30°C. The GST-CDK1/cyclin B was then removed by passing through glutathione sepharose. The bound fraction of glutathione sepharose (contains phosphorylated Ubc9) and flow through fractions (FT, FT2, FT3) were analyzed by 12.5% SDS-PAGE, followed by immunoblotting with anti-GST and anti-Ubc9 antibody. (B) 3 µM, 6 µM and 12 µM of phosphorylated Ubc9 (flow through fraction 3) and non-phosphorylated Ubc9 (flow through fraction 3) were individually incubated with His_6_-SUMO1, SAE1/His_6_-SAE2, GST-hTOP1^(110–125)^ and ATP for 60 min at 37°C. The SUMOylation reaction mixtures were then analyzed by 15% SDS-PAGE, followed by immunoblotting with anti-GST antibody. The amount of phosphorylated and non-phosphorylated Ubc9 for SUMOylation assay were detected by immunoblotting with anti-Ubc9 antibody.

To prove that CDK1/cyclin B-mediated phosphorylation of Ubc9 is responsible for the observed stimulatory effect of CDK1/cyclin B on SUMOylation *in vitro*, His_6_-Ubc9 was pretreated with GST-CDK1/cyclin B in the presence or absence of ATP. After pretreatment, the reaction mixtures were passed through glutathione sepharose three times to remove GST-tagged CDK1/cyclin B (**Figure 3A**). The flow-through fraction 3 (FT3) of phosphorylated and non-phosphorylated His_6_-Ubc9 were subjected to *in vitro* SUMOylation assay. As shown in **Figure 3B**, phosphorylated His_6_-Ubc9 exhibited elevated (about 2-fold) SUMOylation activity as compared to non-phosphorylated His_6_-Ubc9 (**Figure 3B** **; upper panel**). The loading amount of both forms of Ubc9s were relatively equal (**Figure 3B** **; lower panel**). These results suggest that the SUMOylation-stimulatory activity of CDK1/cyclin B is at least in part mediated through its phosphorylation of Ubc9.

### CDK1/cyclin B upregulates SUMOylation by Increasing Thioester Bond Formation between Phosphorylated Ubc9 and SUMO1 but not between SAE1/SAE2 and SUMO1

**Figure 4:**
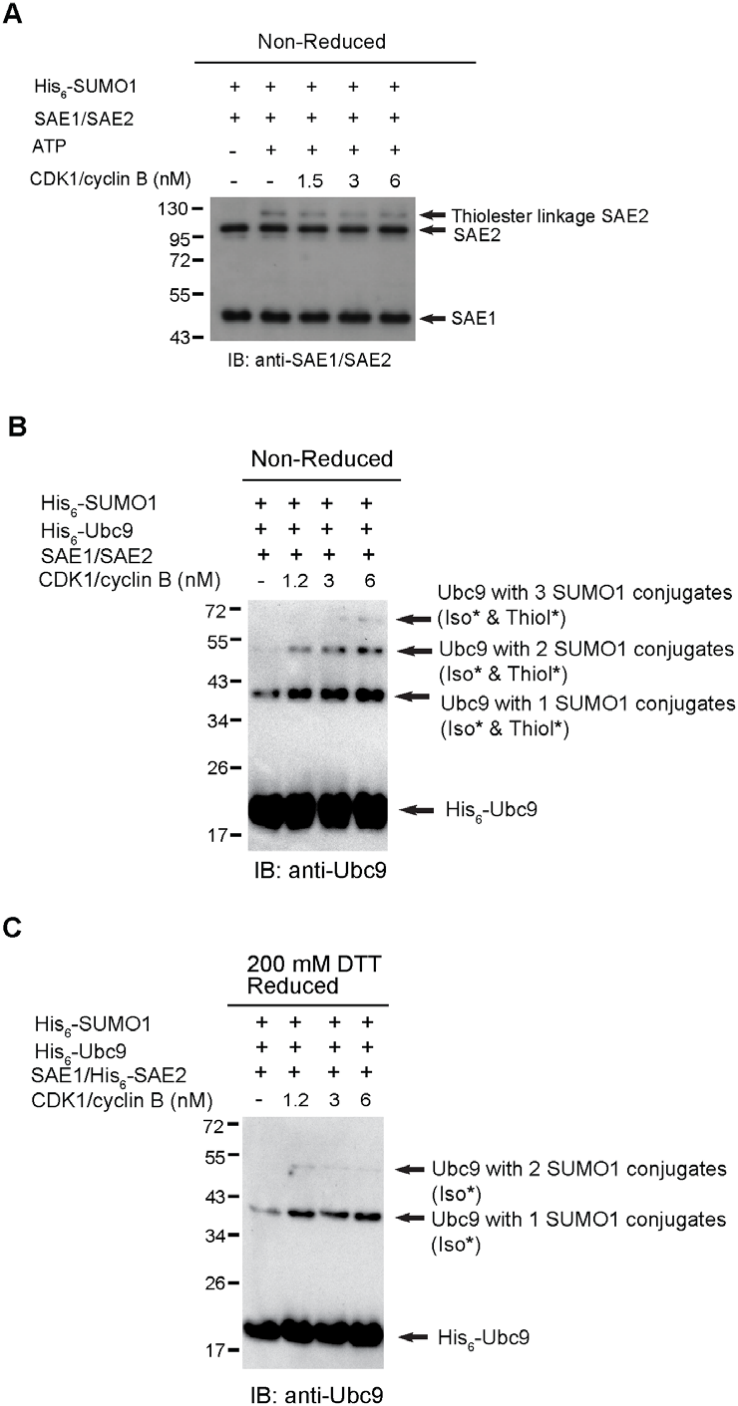
CDK1/cyclin B upregulates SUMOylation by increasing thioester bond formation between phosphorylated Ubc9 and SUMO1 but not between SAE1/SAE2 and SUMO1. (A) To examine the thioester bond formation between His_6_-SUMO1 and SAE1/His_6_-SAE2, purified His_6_-SUMO1 and SAE1/His_6_-SAE2 were incubated with various concentrations of CDK1/cyclin B (1.5 nM, 3 nM and 6 nM) in the presence or absence of ATP. Reaction mixtures were run on a 10% SDS-PAGE and immunoblotted with anti-SAE1/SAE2 antibody. (B) Ubc9 thioester conjugation assay was performed by incubating purified His_6_-Ubc9, SAE1/His_6_-SAE2 and His_6_-SUMO1 with various concentrations of CDK1/cyclin B (1.2 nM, 3 nM and 6 nM) in the presence of ATP for 60 min at 37°C. Samples were then resolved on a 15% SDS-PAGE and probed with anti-Ubc9 antibody. (C) Ubc9 thioester conjugation assay in was performed similar as in (B), but at the end of the reaction, 200 mM of DTT was added to samples for overnight at 40°C. The reduced samples were run on a 15% SDS-PAGE, followed by immunoblotting with anti-Ubc9 antibody. Iso, isopeptide bond; Thiol, thioester bond.

SUMO1 conjugates to E1 activating enzyme (SAE1/SAE2) through thioester bond linkage; whereas, SUMO1 conjugates Ubc9 through thioester and isopeptide bond linkage in the SUMOylation pathway. To examine whether CDK1/cyclin B stimulates SUMOylation through SUMO1 activation of SAE1/SAE2 (in thioester linkage), the SAE1/His_6_-SAE2 and His_6_-SUMO1 were mixed together with increased concentration of CDK1/cyclin B. Result showed that increasing concentration of CDK1/cyclin B did not alter the thioester bond formation between His_6_-SUMO1 and SAE1/His_6_-SAE2 (**Figure 4A**), suggesting that the activation of SUMO1 by SAE1/SAE2 is irrelevant to CDK1/cyclin B treatment. To examine whether CDK1/cyclin B increases SUMOylation through conjugational activity of phosphorylated Ubc9 (in thioester linkage). His_6_-Ubc9, SAE1/His_6_-SAE2 and His_6_-SUMO1 were mixed together with increased concentration of GST-CDK1/cyclin B in the presence or absence of 200 mM DTT. In the non-reduced condition, three different molecular species containing SUMO conjugations in the form of isopeptide and thioester bond were observed. The summation of thioester and isopeptide bond conjugates was greater than the isopeptide bond conjugates as shown in **Figure 4C**. Moreover, the intensity of the summation of thioester and isopeptide bond conjugates was strongly enhanced in a concentration-dependent manner by increased concentration of CDK1/cyclin B (**Figure 4B**). However, due to isopeptide bond formation was not affected by CDK1/cyclin B as shown in **Figure 4C**, therefore, we conclude that thioester bond conjugates was enhanced by CDK1/cyclin B. In the reducing condition, an observation of at least two different molecular species containing either one or two SUMO conjugations in the form of isopeptide bond were observed. This observation agrees with the report that Ubc9 contains multiple SUMO conjugation sites [24], [25]. Moreover, the isopeptide bond conjugations were not enhanced by the increased concentration of CDK1/cyclin B, suggesting that CDK1/cyclin B does not affect the isopeptide bond formation between phosphorylated His_6_-Ubc9 and His_6_-SUMO1 (**Figure 4C**). Altogether, results strongly suggest that CDK1/cyclin B affects Ubc9 function by phosphorylating Ubc9, therefore facilitates and increases thioester bond formation between phosphorylated Ubc9 and SUMO1, ultimately upregulates SUMOylation activity of substrate protein. Additionally, CDK1/cyclin B neither phosphorylates SAE1/SAE2 nor enhances thioester bond formation between SUMO1 and SAE1/SAE2 enzyme.

### CDK1/cyclin B-phosphorylated Ubc9 Stimulates Multi-SUMOylation of Full-length Human Topoisomerase I

**Figure 5:**
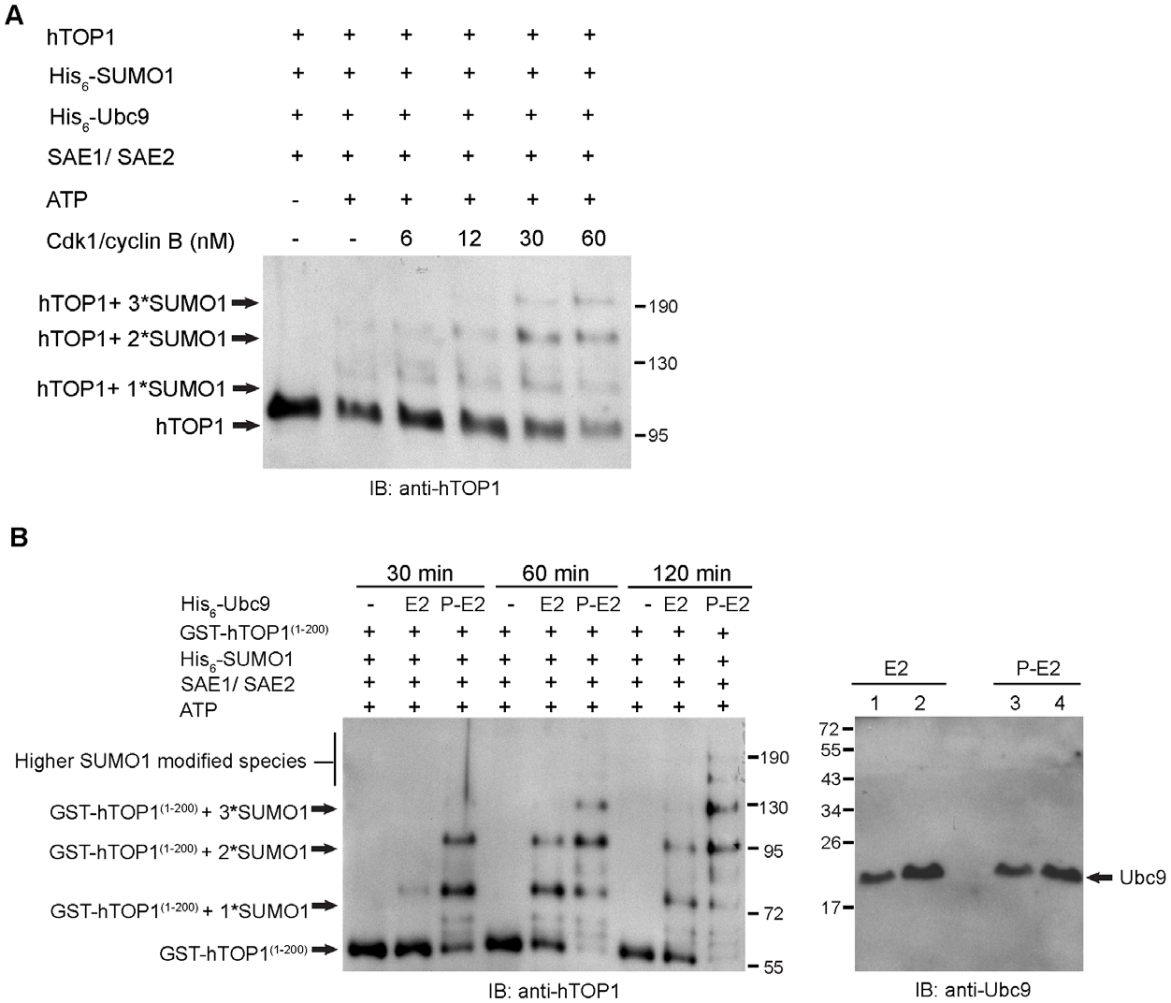
CDK1/cyclin B enhances SUMOylation level of human TOP1 containing multiple SUMO conjugation site. (A) Full-length protein of human topoisomerase I was incubated with His_6_-SUMO1, His_6_-Ubc9, SAE1/His-SAE2 and ATP in the presence of 6, 12, 30 and 60 nM of CDK1/cyclin B. The reaction was resolved on a 6% SDS-PAGE and immunoblotted with anti-topoisomerase I antibody. (B) Equal amount of CDK1/cyclin B phosphorylated Ubc9 (P-E2) and Ubc9 (E2) (6 µM at the final concentration) was individually incubated with His_6_-SUMO1, SAE1/His_6_-SAE2, GST-hTOP1^(1–200)^ and ATP at 37°C for 30 min, 60 min and 120 min. The resulting SUMOylation reaction mixtures were run on a 8% SDS-PAGE, followed by immunoblotting with anti-topoisomerase I antibody. The amount of phosphorylated Ubc9 (P-E2) and Ubc9 (E2) for SUMOylation assay in Fig. 5B was determined by immunoblotting with anti-Ubc9 antibody. Lane 1 and 3 (right panel) were loaded with 1 µg Ubc9 and phosphorylated Ubc9, respectively. Lane 2 and lane 4 (right panel) were loaded with 2 µg of Ubc9 and phosphorylated Ubc9, respectively.

Full-length human topoisomerase I (hTOP1), a cell cycle-regulated protein, contains multiple SUMOylation sites at its N-terminus [25], [22], [26]. Multi-SUMOylation of hTOP1 at its N-terminus occurs both *in vivo* and *in vitro* [25], [22], [26]. hTOP1 also contains multiple phosphorylation sites at its N-terminus including the Ser-112 phosphorylation site that is phosphorylated by CDK1/cyclin B [23]. We therefore sought to determine whether CDK1/cyclin B stimulates multi-SUMOylation of hTOP1 *in vitro* and whether CDK1/cyclin B-phosphorylated Ubc9 is responsible for the stimulation. The substrate used in the *in vitro* system is hTOP1. As shown in **Figure 5A**, CDK1/cyclin B stimulated the formation of multiple high molecular weight species of hTOP1, representing hTOP1 conjugates with one, two and three conjugated SUMO1, suggesting that CDK1/cyclin B stimulates multi-SUMOylation of hTOP1 (**Figure 5A**). We have also investigated the effect of CDK1/cyclin B-phosphorylated Ubc9 on multi-SUMOylation of hTOP1 using GST-hTOP1^(1–200)^ as substrate. As shown in left panel of **Figure 5B**, unphosphorylated Ubc9 stimulated multi-SUMOylation of GST-hTOP1^1–200^ as compared to the control (no Ubc9). Importantly, multi-SUMOylation of GST-hTOP1^(1–200)^ was further stimulated when Ubc9 (E2) was replaced with CDK1/cyclin B-phosphorylated Ubc9 (P-E2) (**Figure 5B** **; left panel**). Higher molecular weight SUMO1-modified species of GST-hTOP1^(1–200)^ were observed together with a concomitant decrease in the level of unmodified GST-hTOP1^(1–200)^ (**Figure 5B** **; left panel**). Because the intensity of Ubc9 and phosphorylated Ubc9 after immunoblotting with anti-Ubc9 antibody is almost the same, which is consistent with Biorad protein assay, thus we use Ubc9 and phosphorylated Ubc9 for SUMOylation assay at a final concentration of 6 µM (**Figure 5B** **; right panel**). These results suggest that CDK1/cyclin B-stimulated multi-SUMOylation of hTOP1 is also likely to be mediated through phosphorylated Ubc9, which provides additional evidence for the possible physiological relevance of CDK1/cyclin B-mediated phosphorylation of Ubc9 in stimulation of SUMOylation.

### CDK1/cyclin B Mediates Phosphorylation of Ubc9 at Serine 71

**Figure 6:**
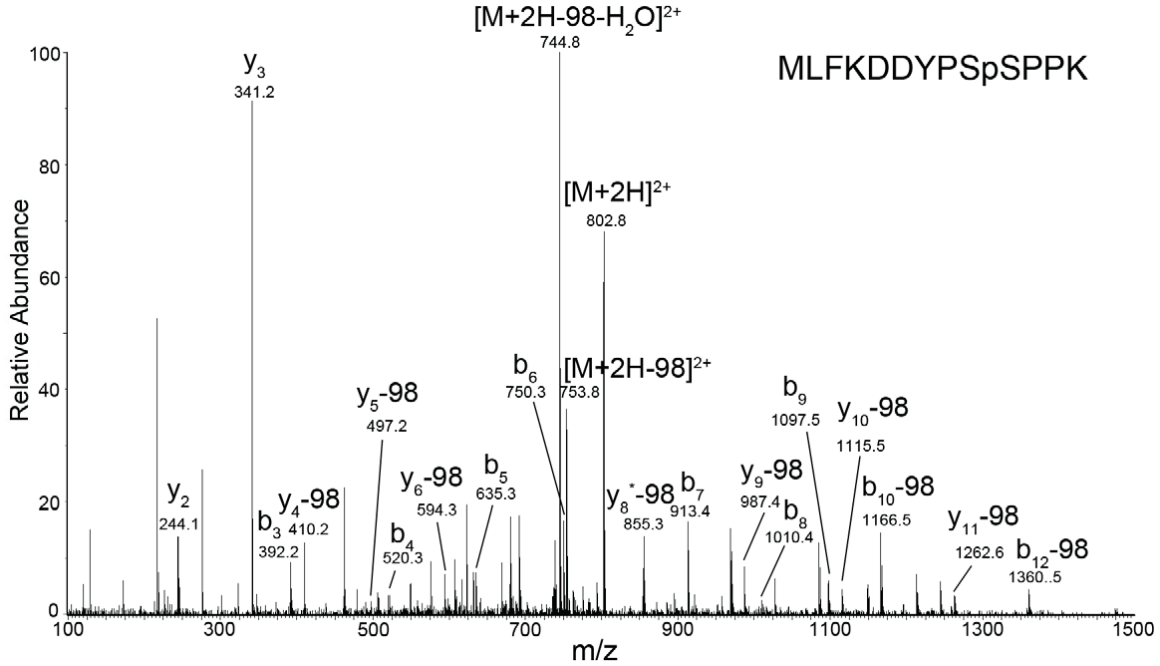
CDK1/cyclin B mediates phopshorylation of Ubc9 at Serine 71. IMAC enriched tryptic peptides of Ubc9 were detected by CID tandem mass spectra. Precursor ion m/z 802.8 (charge state +2) representing the phosphopeptide MLFKDDYPSpSPPK. Residues bearing phosphate moieties are indicated with p. “b” and “y” ions series represent fragment ions containing the N- and C-termini of the peptide, respectively. Product ions marked with * resulted from elimination of NH_3_. The mass of 98 on the peaks was derived from neutral losses (-97.9769 Da) of phosphoric acid (H_3_PO_4_).

In order to identify the phosphorylation sites of Ubc9, *in vitro* phosphorylated as well as non-phosphorylated Ubc9 was first tryptic digested and phosphopeptides were enriched by immobilized metal affinity chromatography (IMAC). Enriched phosphopeptides were then subjected to liquid chromatography coupled with tandem mass spectrometry (LC-MS/MS). As shown in **Figure 6**, the MS/MS analysis revealed a phosphorylation site at serine 71 in a peptide corresponding to residues MLFKDDYPSpSPPK of Ubc9, whereas no phosphorylation was detected at serine 70 (**Figure 6**). The detection of b_9_ ions, with no neutral loss, indicates that the site of phosphorylation is Serine 71. Phosphorylation at 71 is supported through the detection of y_4_-98 and b_10_-98 ions. In addition the phosphate fingerprint of the neutral loss (owing to H_3_PO_4_) from the parent ion is also a positive signal of phosphorylation. In comparison to the negative control, no phosphorylated amino acid residue was detected (**Figure S3**). Overall, these results suggest that Cdk1/cyclin B mediates the phosphorylation of Ubc9 at serine 71.

### CDK1/cyclin B Enhances SUMOylation Activity through Wild Type Ubc9 but not Mutant Ubc9

**Figure 7:**
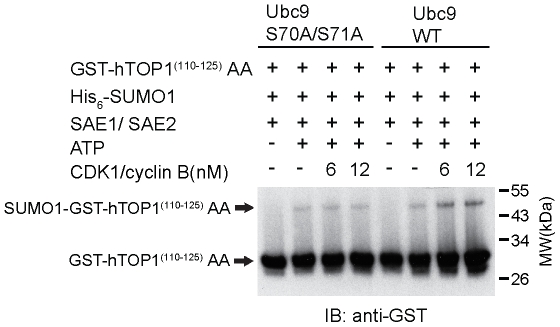
CDK1/cyclin B enhances SUMOylation activity through wild type Ubc9 but not mutant Ubc9 S70A/S71A. Same amount of wild-type and mutant (S70A/S71A) of Ubc9 (at a concentration of 6 µM) was individually added to *in vitro* SUMOylation reaction mixtures containing His_6_-SUMO1, SAE1/His_6_-SAE2, GST-hTOP1 ^(110–125)^ AA and ATP in the presence of CDK1/cyclin B (6 nM and 12 nM) at 37°C for 60 min. The reactions were then boiled in SDS sample buffer and analyzed by 15% SDS-PAGE, followed by immunoblotting with anti-GST antibody.

To determine whether serine 71 plays a role in regulating SUMOylation of Ubc9, serine 70 and serine 71 were mutated to alanine (S70A/S71A), followed by assaying SUMOylation *in vitro*. As predicted, the SUMOylation activity driven by wild-type Ubc9 was enhanced in the presence of CDK1/cyclin B (**Figure 7**). By contrast, SUMOylation activity driven by S70A/S71A mutant Ubc9 was not affected by the addition of CDK1/cyclin B, suggesting that CDK1/cyclin B regulates SUMOylation activity through phosphorylating serine 71 of Ubc9.

## Discussion

The crosstalk between cell cycle and SUMOylation has been widely studied. Our demonstration that CDK1/cyclin B stimulates SUMOylation *in vitro* could indicate a potential molecular link for the crosstalk. CDK1/cyclin B could stimulate SUMOylation through phosphorylation of SUMO target proteins, SUMO machinery components, or a combination of both. In our studies, CDK1/cyclin B was shown to phosphorylate Ubc9 but not SUMO1 or SAE1/SAE2 (**Figure 2**). Our results agree in part with the findings that SUMO machinery components can be phosphorylated and SUMOylated *in vivo* [24], [27]. More importantly, phosphorylation of Ubc9 by CDK1/cyclin B, as demonstrated in our studies, is a novel finding which could have broad implications in cell cycle regulation. Specifically, phosphorylation of Ubc9 could be part of the mitosis regulatory program orchestrated by CDK1/cyclin B.

It is more difficult to determine whether CDK1/cyclin B can also stimulate SUMOylation through its phosphorylation of the SUMO substrate. Hackbarth *et al* suggested that human topoisomerase I contains an *in vivo* phosphorylation site (Ser-112) regulated by CDK1/cyclin B [23]. In our studies, we have confirmed that CDK1/cyclin B indeed phosphorylates GST-hTOP1^(110–125)^ but not GST-hTOP1^(110–125)^AA mutant (Ser 111 and Ser 112 converted to Ala 111 and Ala 112). However, CDK1/cyclin B stimulates SUMOylation of both GST-hTOP1^(110–125)^ and GST-hTOP1^(110–125)^AA mutant to the same extent. These results strongly suggest that the SUMOylation-stimulatory effect of CDK1/cyclin B is due to CDK1/cyclin B-mediated phosphorylation of the SUMOylation machinery (i.e. Ubc9) rather than that of the SUMO substrate (i.e. GST-hTOP1^(110–125)^) in this *in vitro* system. We have also performed *in vitro* SUMOylation assay using a more physiologically relevant substrate, GST-hTOP1^(1–200)^, which contains the entire N-terminus of hTOP1. The N-terminus of hTOP1, which contains multiple CDK1/cyclin B phosphorylation sites, is known to be multi-SUMOylated *in vivo* [22], [26]. Indeed, CDK1/cyclin B stimulates multi-SUMOylation of GST-hTOP1^(1–200)^ in our *in vitro* system. More importantly, CDK1/cyclin B-phosphorylated Ubc9 is much more efficient than unphosphorylated Ubc9 in stimulating multi-SUMOylation of GST-hTOP1^(1–200)^. These results suggest the SUMOylation-stimulatory effect of CDK1/cyclin B is primarily due to its phosphorylation of the SUMOylation machinery (i.e. Ubc9). However, we cannot rule out the possibility that CDK1/cyclin B may also stimulate SUMOylation through its phosphorylation of GST-hTOP1^(1–200)^.

The function of Ubc9 is to conjugate SUMO1 received from SAE1/SAE2. The conjugation requires formation of thioester bond. According to Knipscheer et al., Ubc9 is autoSUMOylated thereby leads to target discrimination in the following SUMO modification, suggesting that Ubc9 itself serves as a substrate and forms isopeptide bond with SUMO on specific lysine [24]. This raises a question whether CDK1/cyclin B enhances thioester bond conjugational activity of Ubc9 with SUMO1 or enhances isopeptide bond linkage of SUMO1 with Ubc9 or substrate protein. Our study showed that the isopeptide bond linkage between CDK1/cyclin B phosphorylated Ubc9 and SUMO1 was unchanged in reduced condition (**Figure 4C**). Conversely, the summation of thioester and isopeptide bond formation between CDK1/cyclin B phosphorylated Ubc9 and SUMO1 was increased in a concentration-dependent manner in non-reduced condition (**Figure 4B**). Because isopeptide bond formation was unaffected by CDK1/cyclin B illustrated in reduced condition, thus thioester bond formation was the only covalent bond to be increased by CDK1/cyclin B. As the amount of thioester bond formation reflects the conjugational capacity of Ubc9, our *in vitro* results clearly indicate that the SUMOylation activity in cell cycle is upregulated when Ubc9 is phosphorylated by CDK1/cyclin B, in addition, the upregulation of SUMOylation activity is directed by increased conjugational activity of phosphorylated Ubc9.

The crosstalk between phosphorylation and SUMOylation has been widely studied in recent years. Notably, many SUMO substrates contain a highly conserved motif having a SUMO consensus site and a proline-directed phosphorylation site (ΨKxExxSP) leading to a concept of phosphorylation-dependent SUMO modification (PDSM) [28]. Thus, phosphorylation has widely been accepted to serve as a regulator for SUMOylation at the substrate level. On the other hand, recent studies have pointed to the possibility that the SUMOylation machinery components may be subject to post-translational modifications. For example, SUMO1 itself has been shown to be phosphorylated *in vivo* by using mass spectrometric approach and Ubc9 is itself SUMO-modified which results in alteration of SUMO target specificity [24], [27]. Our current studies have demonstrated that CDK1/cyclin B can phosphorylate the SUMOylation machinery component Ubc9, adding new evidence for regulation of SUMOylation at the level of SUMOylation machinery.

While our studies implicate Ubc9 phosphorylation and hence SUMOylation to be part of the mitosis regulatory program orchestrated by CDK1/cyclin B, SUMOylation has also been implicated in the regulation other cell cycle phases. For example, SUMO modification of retinoblastoma (Rb) leads to the release of repressive activity of Rb on the E2F transcription factor, resulting in G1/S transition [29]. p73, a member of the p53 family, is SUMO-modified via binding to PIAS-1 during the S phase [30], [31]. Interestingly, CDK1, by partnering with other cyclins, is also known to regulate other cell cycle phases [32], [17]. Therefore, it seems plausible that CDK1, by partnering with different cyclins, could also phosphorylate Ubc9 to enhance its SUMOylation activity that may contribute cell cycle progression at other phases of the cell cycle. Clearly, further studies are necessary to establish the role of Ubc9 phosphorylation in SUMOylation regulation during the cell cycle.

## Materials and Methods

### Plasmid Constructs

Plasmids encoding amino acids 1–97 of SUMO1 (pQE30-SUMO1^(1–97)^), full-length Ubc9 (pQE30-Ubc9), full-length SAE1, full-length SAE2 and full-length human topoisomerase I (hTOP1) have been described previously [22]. DNA encoding hTOP1 fragments which contained single and multiple SUMOylation motifs were subcloned into Glutathione S-transferase (GST) fusion protein *Escherichia coli* expression vector, pGEX-KG [33]. The resulting plasmids are designated pGEX-hTOP1^(110–125)^ and pGEX-hTOP1^(1–200)^, respectively. Plasmid expressing pGEX-hTOP1^(110–125)^AA (Ser 111 and Ser 112 are mutated to Ala 111 and Ala 112, respectively) and plasmid expressing pQE30-Ubc9 S70A/S71A (Ser70 and Ser 71 are mutated to Ala 70 and Ala 71, respectively) were also generated using QuickChange site-directed mutagenesis kit (Stratagene).

### Cell Culture

*Spodoptera frugiperda* Sf21 insect cells (Invitrogen) for the expression of E1 (SAE1/His_6_-SAE2) and full-length hTOP1 were cultured at 27°C in Grace’s medium (Invitrogen) supplemented with 0.33% tissue culture yeastolate (Sigma), 0.33% tissue culture lactalbumin hydrolysate (Sigma), 3% fetal bovine serum and penicillin/streptomycin.

### Protein Expression and Purification

For the expression of SUMO1 and Ubc9, plasmids pQE30-SUMO1^(1–97)^ and, pQE30-Ubc9 and pQE30-Ubc9 S70A/S71A were transformed into *E.coli* TOP10 strain. The detailed procedure for expression and purification has been described previously [22]. pGEX-hTOP1^(110–125)^, pGEX-hTOP1^(1–200)^ and pGEX-hTOP1^(110–125)^ AA were individually transformed into *E.coli* TOP10 strain. Transformants were inoculated into LB broth containing ampicillin (50 <$>\raster="rg1"<$>g/ml). Isopropyl β-D-1-thiogalactopyranoside (IPTG) was added to a final concentration of 0.4 mM, when A_600_ nm reached OD 0.3. The culture was incubated for another 2h at 37°C. Cells were centrifuged at 4000 xg for 30 min and resuspended in 1x PBS buffer (140 mM NaCl, 2.7 mM KCl, 10 mM Na_2_HPO_4_, 1.8 mM KH_2_PO_4_, pH 7.3). 1 mM PMSF and 1 mg/ml lysozyme were added to cell suspensions followed by sonication. Cell lysates were then centrifuged at 13,000 xg for 30 min. Supernatants were passed through the Glutathione Sepharose 4B column (bed volume, 1 ml). Nonspecific proteins were washed out with 1 x PBS multiple times until no protein was detected by Biorad protein assay. GST-hTOP1^(110–125)^ and GST-hTOP1^(110–125)^AA were individually eluted with glutathione elution buffer (50 mM Tris-HCl, pH 7.4; 10 mM reduced glutathione, pH 8.0). For baculovirus expression and purification, recombinant SAE1/His_6_-SAE2 and full-length hTOP1 were expressed and purified as described previously [22].

### *In vitro* SUMOylation Assays

To study the effect of various kinases on SUMOylation, His_6_-SUMO1 (1 µg), His_6_-Ubc9 (1 µg), SAE1/His_6_-SAE2 (100 ng), substrate protein [GST-hTOP1^(110–125)^, GST-hTOP1^(1–200)^, GST-hTOP1^(110–125)^AA or full-length hTOP1 (0.5 µg)], ATP (5 mM) and SUMO reaction buffer (20 mM HEPES, pH 7.5; 5 mM MgCl_2_) were mixed together in a total reaction volume of 20 µl. The reaction mixtures were incubated with various concentrations of individual recombinant kinases such as CDK1/cyclin B (6 nM, 12 nM and 30 nM) (Cell Signaling Technology & New England Biolabs), CDK2/cyclin E (5 nM, 9 nM and 23 nM) (Cell Signaling Technology), ERK1 (7 nM, 14 nM and 35 nM) and ERK2 (7 nM, 15 nM and 37 nM) (Millipore) and JNK2/SAPK1 (11 nM, 22 nM and 56 nM) (Millipore) at 37°C for 60 min, followed by boiling in SDS sample buffer for 10 min. Samples were then analyzed by SDS-PAGE and immunoblotted with goat anti-GST antibody (Santa Cruz) or anti-topoisomerase I antibody (LAE Biotech).

To study the concentration-dependent effect of phosphorylated Ubc9 on SUMOylation activity, GST-hTOP1^(110–125)^ (0.5 µg) was incubated with His_6_-SUMO1 (1 µg), SAE1/His_6_-SAE2 (100 ng), ATP (5 mM) and reaction buffer (20 mM HEPES, pH 7.5, 5 mM MgCl_2_) in the presence of increasing concentrations of phosphorylated or non-phosphorylated Ubc9 (3 µM, 6 µM and 12 µM) in a total volume of 20 µl. The reaction mixture was incubated at 37°C for 60 min and then boiled in SDS sample buffer for 10 min. To study the time course effect, GST-hTOP1^(1–200)^ (0.5 µg) was incubated with His_6_-SUMO1 (1 µg), SAE1/His_6_-SAE2 (100 ng), ATP (5 mM) and reaction buffer (20 mM HEPES, pH 7.5, 5 mM MgCl_2_) in the presence of phosphorylated or non-phosphorylated Ubc9 (6 µM). The reaction mixture was incubated at 37°C for three various time points (30 min, 60 min and 120 min), followed by boiling in SDS sample buffer for 10 min. All heat-denatured samples were analyzed by SDS-PAGE and immunoblotted with goat anti-GST antibody (Santa Cruz) or anti-topoisomerase I antibody (LAE Biotech).

To study if Cdk1/cyclin B affects SUMOylation driven by mutant Ubc9, wild type or S70A/S71A mutant Ubc9 (6 µM at final concentration) was incubated with His_6_-SUMO1 (1 µg), SAE1/His_6_-SAE2 (100 ng), GST-hTOP1^(110–125)^AA, ATP (5 mM) and SUMO reaction buffer (20 mM HEPES, pH 7.5; 5 mM MgCl_2_) in the presence of CDK1/cyclin B (6 nM and 12 nM) (New England Biolabs) in a total reaction volume of 20 µl at 37°C for 60 min.

### *In vitro* Phosphorylation Assays

The phosphorylation assay was performed in 20 µl of reaction mixtures. SAE1/His_6_-SAE2 (1 µg) was incubated with various concentrations (3 nM, 6 nM and 12 nM) of CDK1/cyclin B in the presence of [γ-^32^P] ATP (0.05 µCi/µl) in Protein kinase buffer provided by supplier (New England Biolabs). CDK1/cyclin B (3 nM, 6 nM and 12 nM) were also incubated with [γ-^32^P] ATP (0.05 µCi/µl) in protein kinase buffer in the presence or absence of His_6_-SUMO1 (1 µg), His_6_-Ubc9 (1 µg) or GST-hTOP1^(110–125)^ AA (1 µg). All reaction mixtures were incubated at 30°C for 30 min and boiled in SDS sample buffer for 10 min. Samples were then analyzed by SDS-PAGE followed by Coomassie blue staining and autoradiography.

### Isolation of Phosphorylated Ubc9

GST-CDK1/cyclin B from Cell Signaling Technology (35 nM) was incubated with His_6_-Ubc9 (10 µg) in a 200 µl phosphorylation reaction mixture containing CDK1/cyclin B kinase buffer in the presence or absence of ATP (5 mM) at 30°C for 30 min. The reaction mixture was passed through Glutathione sepharose 4B (200 µl bed volume) to remove the GST-tagged CDK1/cyclin B from the mixture. For checking the integrity of phosphorylated Ubc9, the flow-through was analyzed by SDS-PAGE and immunoblotted with goat anti-GST (GE Healthcare) and goat anti-Ubc9 antibodies (Santa Cruz).

### Thioester Conjugation Assay

To examine whether CDK1/cyclin B affects thioester bond formation between SUMO1 and SAE1/SAE2, reaction mixture containing His_6_-SUMO1 (1 µg), SAE1/His_6_-SAE2 (100 ng), SUMO reaction buffer (20 mM HEPES, pH 7.5; 5 mM MgCl_2_) and ATP (5 mM) were incubated with various concentrations (1.5 nM, 3 nM and 6 nM) of CDK1/cyclin B in a total volume of 20 µl at 37°C for 60 min. Reactions were heat-denatured with sample buffer without ß-mercaptoethanol or DTT for 10 min and resolved on SDS-PAGE and immunoblotting with rabbit anti-SAE1/SAE2 antibody.

To examine whether thioester conjugational activity of Ubc9 is affected by CDK1/cyclin B, reaction mixture containing His_6_-Ubc9 (1 µg), His_6_-SUMO1 (1 µg) and SAE1/His_6_-SAE2 (100 ng) were incubated with increased concentration (1.2 nM, 3 nM and 6 nM) of CDK1/cyclin B in a total reaction volume of 20 µl at 37°C for 60 min. The reactions were prepared in two sets, one set of the reaction samples were reduced with DTT (200 mM) and the other set was remained in non-reduced at 40°C for overnight. The intramolecular noncovalent bonds of both reaction samples were heat-denatured for 10 min with urea (8 M) and sample buffer without ß-mercaptoethanol or DTT. The samples were resolved on SDS-PAGE and immunoblotted with goat anti-Ubc9 antibody (Santa Cruz).

### In-solution Digestion and Phosphopeptide Enrichment

To identify the phosphorylation site of Ubc9 which is phosphorylated by CDK1/cyclin B, a solution-based *in vitro* phosphorylation of Ubc9 was initially prepared, the end-reaction of Ubc9 phosphorylation was firstly reduced with 10 mM DTT at room temperature for 60 min. Alkylation reaction was then carried out by adding 55 mM iodoacetanide at room temperature for 20 min. Reduced and alkylated Ubc9 was subsequently digested with trypsin 37°C overnight. Resulting tryptic Ubc9 phopshopeptides were subjected to phosphopeptide enrichment procedures supplied by Clontech. Enriched Ubc9 phosphopeptides were acidified TFA for further ZipTips^TM^ desalting procedure (Millipore).

### LC-ESI-MS/MS Analyses

Peptide samples were reconstituted in buffer A (0.1% FA in H2O) and analyzed on Waters Synapt G2 HDMS (Waters, Milford, MA). Samples were injected onto a 2 cm×180 µm capillary trap column and separated on a 75 µm×5 cm nanoACQUITY 1.7 µm BEH C18 column using a nanoACQUITY Ultra-Performance LC system (Waters, Milford, MA). The column was maintained at 35°C and bound peptides were eluted with a gradient of 5–40% buffer B (buffer A, 0.1% FA in H2O; buffer B, 0.1% FA in ACN) for 30 min. The MS was operated in ESI positive V mode with a resolving power of 10000 and calibrated with a synthetic human [Glu1]-Fibrinopeptide B solution (0.5 pmol/mL, Sigma-Aldrich) delivered through the NanoLockSpray source. Data acquisition was performed using data directed analysis (DDA) method. The DDA method included one full MS scan (m/z 350–1600, 1 s/scan) and three MS/MS scans (m/z 100–2000, 1 s/scan) performed sequentially on the three most intense ions present in the full scan mass spectrum.

## Supporting Information

Figure S1 **Determination of the phosphorylation activities of different kinases.** Various concentrations of (A) CDK1/cyclin B (12 nM, 30 nM and 60 nM) or (B) CDK2/cyclin E (9 nM, 23 nM and 46 nM) were incubated with (left) or without (right) histone H1 for 30 min at 30°C in the presence of [γ-^32^P] ATP. The reactions were analyzed by 12.5% SDS-PAGE followed by Coomassie blue staining and autoradiography. Various concentrations of (C) ERK1 (14 nM, 35 nM and 71 nM), (D) ERK2 (15 nM, 37 nM and 74 nM) or (E) PKA (24 nM, 61 nM and 122 nM) was incubated with (left) or without (right) myelin basic protein (MBP) for 30 min at 30°C in the presence of [γ-^32^P] ATP. The reactions were analyzed by 15% SDS-PAGE followed by Coomassie blue staining and autoradiography. Various concentrations of (F) JNK2/SAPK1 (22 nM, 56 nM and 111 nM) was incubated with (left) or without (right) activating transcription factor 2 (ATF2) for 30 min at 30°C in the presence of [γ-^32^P] ATP. The reaction was analyzed by 10% SDS-PAGE followed by Coomassie blue staining and autoradiography.
(TIF)

Figure S2 **Determination of the phosphorylation activity of CDK1/cyclin B.** Twelve nM of CDK1/cyclin B was incubated with (left) or without (right) histone H1 for 30 min at 30°C in the presence of [γ-^32^P] ATP (0.05 µCi/µl). The reactions were analyzed by 12.5% SDS-PAGE followed by Coomassie blue staining and autoradiography.
(TIF)

Figure S3 **Non-phosphorylated Ubc9 as a negative control analyzed by CID tandem** **mass spectra.** Precursor ion m/z 508.92 (charge state +3) representing the peptide MLFKDDYPSSPPK. ″b″ and ″y″ ions series represent fragment ions containing the N- and C-termini of the peptide, relatively.
(TIF)

Text S1 **Supporting methods.**
(DOC)

## References

[1] Hay RT (2005). SUMO: a history of modification.. Mol Cell.

[2] Azuma Y, Arnaoutov A, Dasso M (2003). SUMO-2/3 regulates topoisomerase II in mitosis.. J Cell Biol.

[3] Fernandez-Miranda G, de Castro IP, Carmena M, Aguirre-Portoles C, Ruchaud S (2010). SUMOylation modulates the function of Aurora-B kinase.. J Cell Sci 123 (Pt.

[4] Seufert W, Futcher B, Jentsch S (1995). Role of a ubiquitin-conjugating enzyme in degradation of S- and M-phase cyclins.. Nature.

[5] al-Khodairy F, Enoch T, Hagan IM, Carr AM (1995). The Schizosaccharomyces pombe hus5 gene encodes a ubiquitin conjugating enzyme required for normal mitosis.. J Cell Sci 108 ( Pt.

[6] Nacerddine K, Lehembre F, Bhaumik M, Artus J, Cohen-Tannoudji M (2005). The SUMO pathway is essential for nuclear integrity and chromosome segregation in mice.. Dev Cell.

[7] Nowak M, Hammerschmidt M (2006). Ubc9 regulates mitosis and cell survival during zebrafish development.. Mol Biol Cell.

[8] Biggins S, Bhalla N, Chang A, Smith DL, Murray AW (2001). Genes involved in sister chromatid separation and segregation in the budding yeast Saccharomyces cerevisiae.. Genetics.

[9] Tanaka K, Nishide J, Okazaki K, Kato H, Niwa O (1999). Characterization of a fission yeast SUMO-1 homologue, pmt3p, required for multiple nuclear events, including the control of telomere length and chromosome segregation.. Mol Cell Biol.

[10] Joseph J, Tan SH, Karpova TS, McNally JG, Dasso M (2002). SUMO-1 targets RanGAP1 to kinetochores and mitotic spindles.. J Cell Biol.

[11] Li SJ, Hochstrasser M (1999). A new protease required for cell-cycle progression in yeast.. Nature.

[12] Denison C, Rudner AD, Gerber SA, Bakalarski CE, Moazed D (2005). A proteomic strategy for gaining insights into protein sumoylation in yeast.. Mol Cell Proteomics.

[13] Bachant J, Alcasabas A, Blat Y, Kleckner N, Elledge SJ (2002). The SUMO-1 isopeptidase Smt4 is linked to centromeric cohesion through SUMO-1 modification of DNA topoisomerase II.. Mol Cell.

[14] Wohlschlegel JA, Johnson ES, Reed SI, Yates (2004). Global analysis of protein sumoylation in Saccharomyces cerevisiae.. J Biol Chem.

[15] Azuma Y, Arnaoutov A, Anan T, Dasso M (2005). PIASy mediates SUMO-2 conjugation of Topoisomerase-II on mitotic chromosomes.. EMBO J.

[16] Losada A, Hirano T (2005). Dynamic molecular linkers of the genome: the first decade of SMC proteins.. Genes Dev.

[17] Satyanarayana A, Kaldis P (2009). Mammalian cell-cycle regulation: several Cdks, numerous cyclins and diverse compensatory mechanisms.. Oncogene.

[18] Blangy A, Lane HA, d'Herin P, Harper M, Kress M (1995). Phosphorylation by p34cdc2 regulates spindle association of human Eg5, a kinesin-related motor essential for bipolar spindle formation in vivo.. Cell.

[19] Peter M, Nakagawa J, Doree M, Labbe JC, Nigg EA (1990). In vitro disassembly of the nuclear lamina and M phase-specific phosphorylation of lamins by cdc2 kinase.. Cell.

[20] Kimura K, Cuvier O, Hirano T (2001). Chromosome condensation by a human condensin complex in Xenopus egg extracts.. J Biol Chem.

[21] Rudner AD, Murray AW (2000). Phosphorylation by Cdc28 activates the Cdc20-dependent activity of the anaphase-promoting complex.. J Cell Biol.

[22] Yang M, Hsu CT, Ting CY, Liu LF, Hwang J (2006). Assembly of a polymeric chain of SUMO1 on human topoisomerase I in vitro.. J Biol Chem.

[23] Hackbarth JS, Galvez-Peralta M, Dai NT, Loegering DA, Peterson KL (2008). Mitotic phosphorylation stimulates DNA relaxation activity of human topoisomerase I. J Biol Chem.

[24] Knipscheer P, Flotho A, Klug H, Olsen JV, van Dijk WJ (2008). Ubc9 sumoylation regulates SUMO target discrimination.. Mol Cell.

[25] Hsiao HH, Meulmeester E, Frank BT, Melchior F, Urlaub H (2009). "ChopNSpice," a mass spectrometric approach that allows identification of endogenous small ubiquitin-like modifier-conjugated peptides.. Mol Cell Proteomics.

[26] Hwong CL, Chen MS, Hwang JL (1989). Phorbol ester transiently increases topoisomerase I mRNA levels in human skin fibroblasts.. J Biol Chem.

[27] Matic I, Macek B, Hilger M, Walther TC, Mann M (2008). Phosphorylation of SUMO-1 occurs in vivo and is conserved through evolution.. J Proteome Res.

[28] Hietakangas V, Anckar J, Blomster HA, Fujimoto M, Palvimo JJ (2006). PDSM, a motif for phosphorylation-dependent SUMO modification.. Proc Natl Acad Sci U S A.

[29] Ledl A, Schmidt D, Muller S (2005). Viral oncoproteins E1A and E7 and cellular LxCxE proteins repress SUMO modification of the retinoblastoma tumor suppressor.. Oncogene.

[30] Munarriz E, Barcaroli D, Stephanou A, Townsend PA, Maisse C (2004). PIAS-1 is a checkpoint regulator which affects exit from G1 and G2 by sumoylation of p73.. Mol Cell Biol.

[31] Gaiddon C, Lokshin M, Gross I, Levasseur D, Taya Y (2003). Cyclin-dependent kinases phosphorylate p73 at threonine 86 in a cell cycle-dependent manner and negatively regulate p73.. J Biol Chem.

[32] Santamaria D, Barriere C, Cerqueira A, Hunt S, Tardy C (2007). Cdk1 is sufficient to drive the mammalian cell cycle.. Nature.

[33] Chen TY, Hsu CT, Chang KH, Ting CY, Whang-Peng J (2000). Development of DNA delivery system using Pseudomonas exotoxin A and a DNA binding region of human DNA topoisomerase I. Appl Microbiol Biotechnol.

