# Supplementary figures and images for: Phosphorylation of Ubc9 by Cdk1 Enhances SUMOylation Activity
Source: PLoS One. 2012 Apr 3;7(4):e34250. doi: 10.1371/journal.pone.0034250 (PMC3317942; doi:10.1371/journal.pone.0034250)

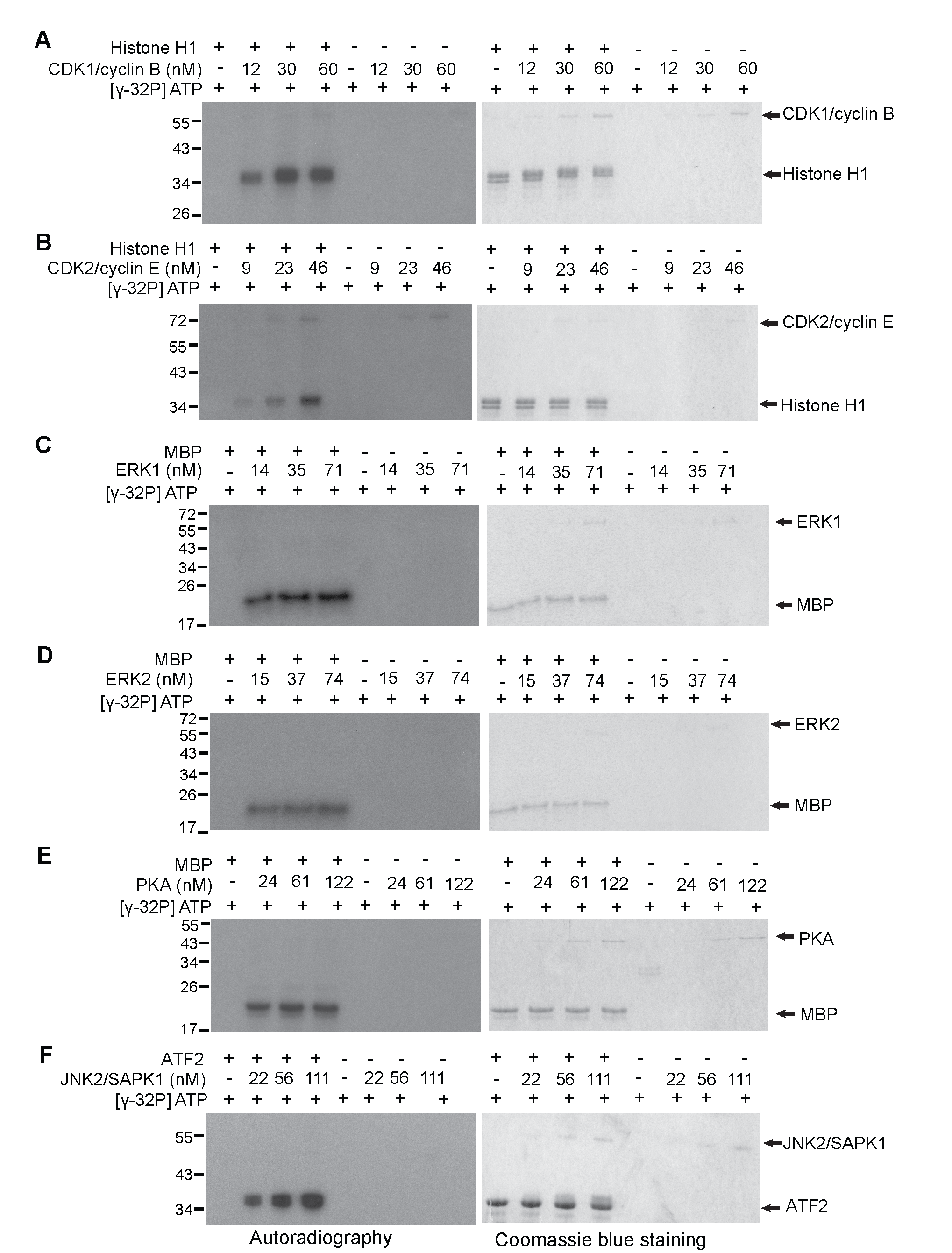

Supplement: Figure S1 — Determination of the phosphorylation activities of different kinases. Various concentrations of (A) CDK1/cyclin B (12 nM, 30 nM and 60 nM) or (B) CDK2/cyclin E (9 nM, 23 nM and 46 nM) were incubated with (left) or without (right) histone H1 for 30 min at 30°C in the presence of [γ-32P] ATP. The reactions were analyzed by 12.5% SDS-PAGE followed by Coomassie blue staining and autoradiography. Various concentrations of (C) ERK1 (14 nM, 35 nM and 71 nM), (D) ERK2 (15 nM, 37 nM and 74 nM) or (E) PKA (24 nM, 61 nM and 122 nM) was incubated with (left) or without (right) myelin basic protein (MBP) for 30 min at 30°C in the presence of [γ-32P] ATP. The reactions were analyzed by 15% SDS-PAGE followed by Coomassie blue staining and autoradiography. Various concentrations of (F) JNK2/SAPK1 (22 nM, 56 nM and 111 nM) was incubated with (left) or without (right) activating transcription factor 2 (ATF2) for 30 min at 30°C in the presence of [γ-32P] ATP. The reaction was analyzed by 10% SDS-PAGE followed by Coomassie blue staining and autoradiography. (TIF) [file pone.0034250.s001.tif]

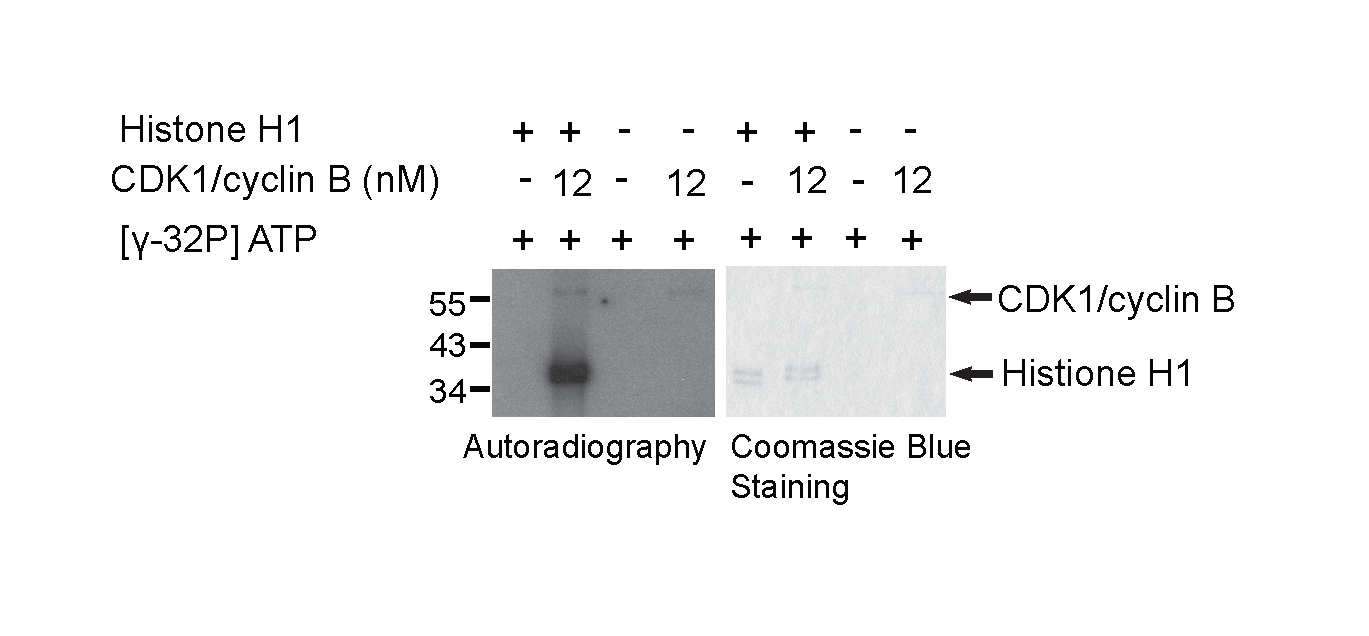

Supplement: Figure S2 — Determination of the phosphorylation activity of CDK1/cyclin B. Twelve nM of CDK1/cyclin B was incubated with (left) or without (right) histone H1 for 30 min at 30°C in the presence of [γ-32P] ATP (0.05 µCi/µl). The reactions were analyzed by 12.5% SDS-PAGE followed by Coomassie blue staining and autoradiography. (TIF) [file pone.0034250.s002.tif]

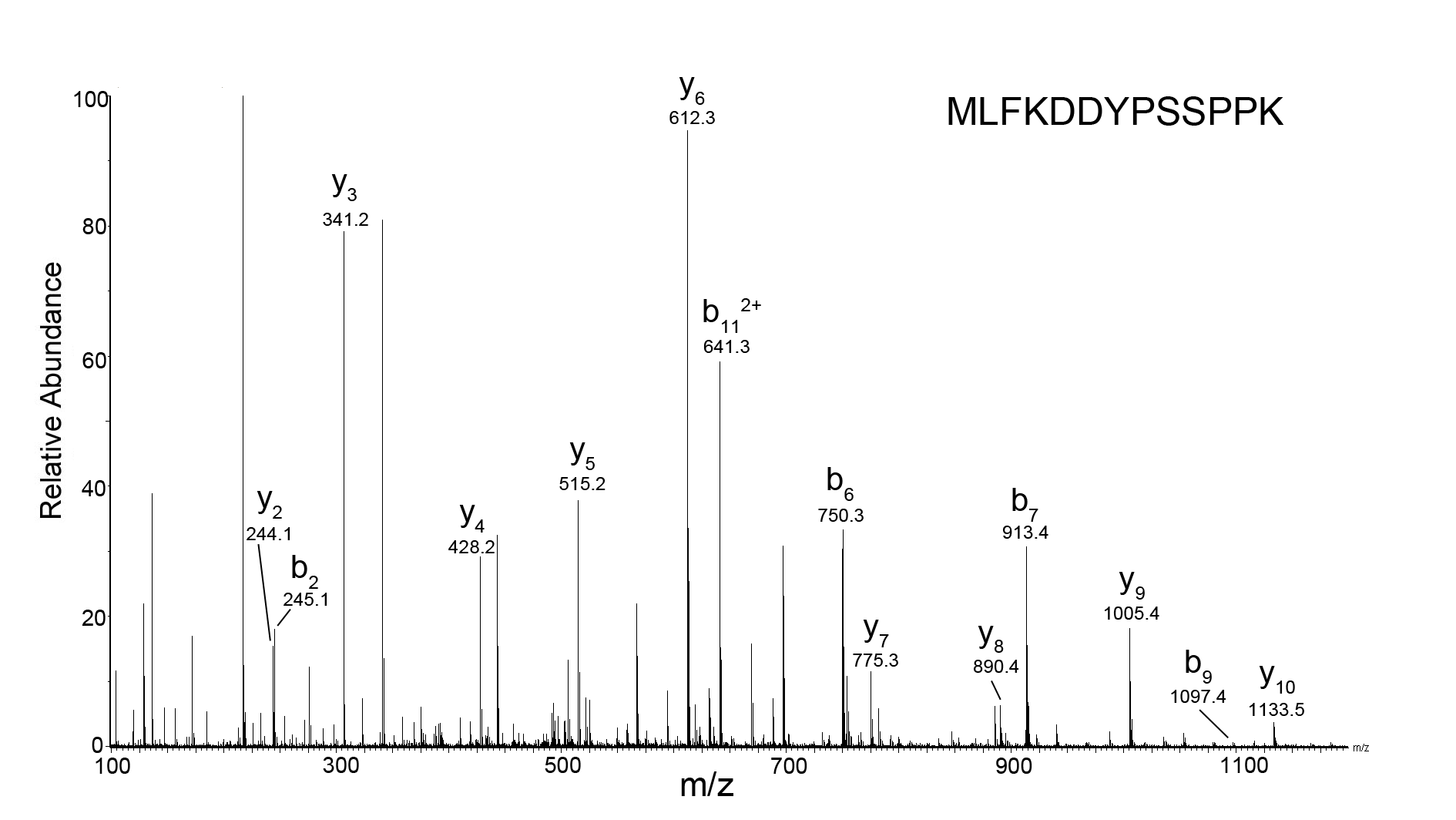

Supplement: Figure S3 — Non-phosphorylated Ubc9 as a negative control analyzed by CID tandem mass spectra. Precursor ion m/z 508.92 (charge state +3) representing the peptide MLFKDDYPSSPPK. ″b″ and ″y″ ions series represent fragment ions containing the N- and C-termini of the peptide, relatively. (TIF) [file pone.0034250.s003.tif]
