# Supplementary material for: Phosphorylation of Ubc9 by Cdk1 Enhances SUMOylation Activity
Source: PLoS One. 2012 Apr 3;7(4):e34250. doi: 10.1371/journal.pone.0034250 (PMC3317942; doi:10.1371/journal.pone.0034250)
Supplement: Text S1 — Supporting methods. (DOC) [file pone.0034250.s004.doc]

**Supporting methods**

*In vitro* phosphorylation assays

The phosphorylation assay was performed in 20 μl of reaction mixtures. Recombinant substrate protein histone H1 (1 μg) (Milipore), was incubated in with various concentrations of CDK1/cyclin B (12 nM, 30 nM and 60 nM) or CDK2/cyclin E (9 nM, 23 nM and 46 nM) in the presence of [γ-^32^P] ATP (0.05 μCi/μl) in kinase buffer. Myelin basic protein (MBP) (1 μg) (Milipore) was incubated in with various concentrations of ERK1 (14 nM, 35 nM and 71 nM) or ERK2 (15 nM, 37 nM and 74 nM) or PKA (24 nM，61 nM and 122 nM) in the presence of [γ-^32^P] ATP (0.05 μCi/μl) in kinase buffer. Activating transcription factor 2 (ATF2) was incubated with various concentrations (22 nM, 56 nM and 111 nM) of JNK2/SAPK1 in the presence of [γ-^32^P] ATP (0.05 μCi/μl) in kinase buffer. All reaction mixtures were incubated at 30°C for 30 min and boiled in SDS sample buffer for 10 min. Samples were then analyzed by SDS-PAGE followed by Coomassie blue staining and autoradiography.
